# Clustering ionic flow blockade toggles with a Mixture of HMMs

**DOI:** 10.1186/1471-2105-9-S9-S13

**Published:** 2008-08-12

**Authors:** Alexander Churbanov, Stephen Winters-Hilt

**Affiliations:** 1 The Research Institute for Children, 200 Henry Clay Ave., New Orleans, LA 70118, USA; 2 Department of Computer Science, University of New Orleans, New Orleans, LA, 70148, USA

## Abstract

**Background:**

Ionic current blockade signal processing, for use in nanopore detection, offers a promising new way to analyze single molecule properties with potential implications for DNA sequencing. The *α*-Hemolysin transmembrane channel interacts with a translocating molecule in a nontrivial way, frequently evidenced by a complex ionic flow blockade pattern with readily distinguishable modes of toggling. Effective processing of such signals requires developing machine learning methods capable of learning the various blockade modes for classification and knowledge discovery purposes. Here we propose a method aimed to improve our stochastic analysis capabilities to better understand the discriminatory capabilities of the observed the nanopore channel interactions with analyte.

**Results:**

We tailored our memory-sparse distributed implementation of a Mixture of Hidden Markov Models (MHMMs) to the problem of channel current blockade clustering and associated analyte classification. By using probabilistic fully connected HMM profiles as mixture components we were able to cluster the various 9 base-pair hairpin channel blockades. We obtained very high Maximum a Posteriori (MAP) classification with a mixture of 12 different channel blockade profiles, each with 4 levels, a configuration that can be computed with sufficient speed for real-time experimental feedback. MAP classification performance depends on several factors such as the number of mixture components, the number of levels in each profile, and the duration of a channel blockade event. We distribute Baum-Welch Expectation Maximization (EM) algorithms running on our model in two ways. A distributed implementation of the MHMM data processing accelerates data clustering efforts. The second, simultanteous, strategy uses an EM checkpointing algorithm to lower the memory use and efficiently distribute the bulk of EM processing in processing large data sequences (such as for the progressive sums used in the HMM parameter estimates).

**Conclusion:**

The proposed distributed MHMM method has many appealing properties, such as precise classification of analyte in real-time scenarios, and the ability to incorporate new domain knowledge into a flexible, easily distributable, architecture. The distributed HMM provides a feature extraction that is equivalent to that of the sequential HMM with a speedup factor approximately equal to the number of independent CPUs operating on the data. The MHMM topology learns clusters existing within data samples via distributed HMM EM learning. A Java implementation of the MHMM algorithm is available at .

## Background

The bacterium *Staphylococcus aureus *secretes *α*-hemolysin monomers that bind to the outer membrane of susceptible cells. Seven monomers can oligomerize to form a very stable water-filled transmembrane channel [1]. The channel can cause death to the target cell by rapidly discharging vital molecules (such as ATP) and disturbing the membrane potential.

**Figure 1:**
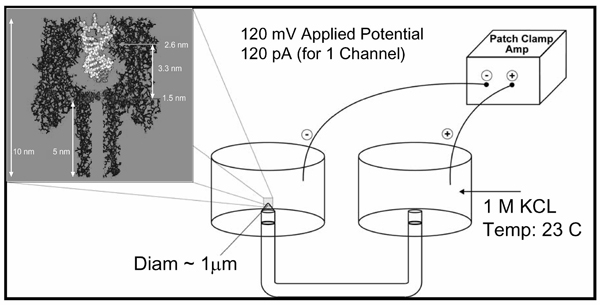
*α*-hemolysin nanopore with captured hairpin.

**Figure 2:**
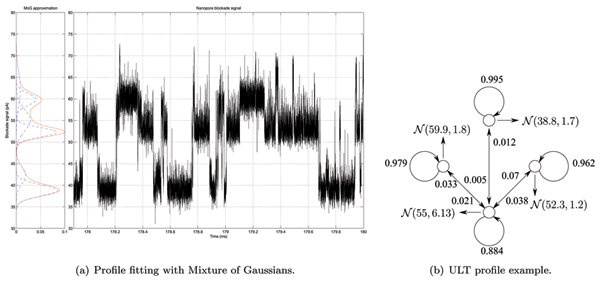
Upper Level Toggler (ULT) with profile example.

Suspended in lipid bilayer, as shown in Figure 1, the *α*-hemolysin channel can be used as a sensor (nanopore-detector) when large molecules interact with the channel environment under an applied potential (where the open channel has 120 picoAmperes of ion flow under normal conditions). When a 9 bp DNA hairpin enters the pore, the loop is caught at the vestibule mouth, leaving the stem terminus perched to readily bind to the amino acid residues near the limiting aperture, resulting in a consistent toggle for thousands of milliseconds as shown in Figure 2.

**Figure 3:**
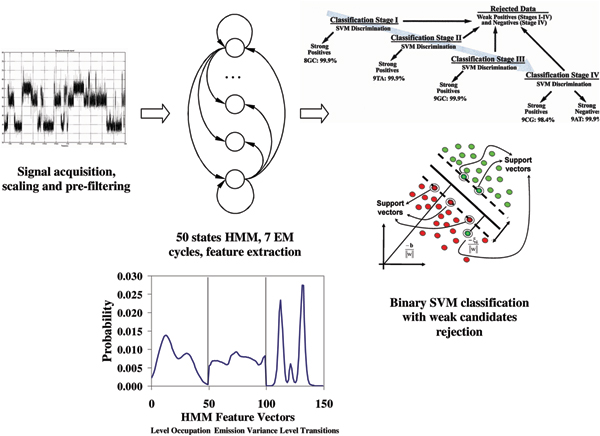
Existing classification process with HMM feature extraction followed by SVM binary tree decision.

Many approaches to characterizing of nucleic acid analyte – channel interactions use 2-D scatter plot analysis [2,3]. A recently proposed method of discriminating translocating RNA polynucleotide orientation [4] uses a combination of six sigmoid phenomenological functional forms to approximate possible blockades. A hybrid method of automated analyte classification was used in [5,6] that discriminates among 8GC, 9GC, 9CG, 9TA and 9AT molecules by first obtaining features extracted with Expectation Maximization (EM) learning on a *single *50-state fully connected Hidden Markov Model (HMM). They then construct a feature vector based on the HMM parameters and pass that to a Support Vector Machine (SVM) for classification (with the binary decision tree shown in Figure 3). Although the process shown in Fig. 3 is scalable, and has high classification accuracy, it can also involve high data rejection rates (good for performing solution assays). This motivates effort to have a less scalable, but lower data-rejection rate (such as what is needed during genomic sequencing). Later study of the data examined in [5,6], with PCA reduction on states followed by a simple, uninformed, AdaBoost classification (not SVM, see [7]), led to similar improvement on zero-rejection accuracy, and thus similar improvements (reductions) on the data-rejection needed for high-accuracy classification [7]. That approach, however, didn't begin with the stronger (but non-scalable in class-number) feature extraction method described here. This is the first test of what is expected to be a highly accurate feature extraction method (better than those employed previously), where the critical limitation in general use, however, is in its scalability in number of classes to discriminate.

In an interview [In Focus, January 2002], one of the pioneers in the development of nanopore technology, Dr. Mark Akeson, states that getting a machine to learn base pair or nucleotide signatures and report the results automatically will be a key feature of a nanopore sequencing instrument. Here we propose a new method of unsupervised learning of ionic flow blockades with Mixture of Hidden Markov Models (MHMM) profiles that has a number of attractive attributes, at the expense of restricting learning to a smaller state space. For genome sequencing, the problem reduces to identifying the classes {*A*, *C*, *G*, *T*}, i.e., there are only four classes to discriminate. Thus, for some important problems the non-scaling constraint is not an issue and this approach may offer the best performance.

The Maximum a Posteriori (MAP) molecular classification with our model opens the possibility for making distributed decisions in real time. The EM algorithms running on our model are computationally expensive procedures, thus, an important method in this work involves computational speed-up efforts via distributed processing implementations.

## Results

**Figure 4:**
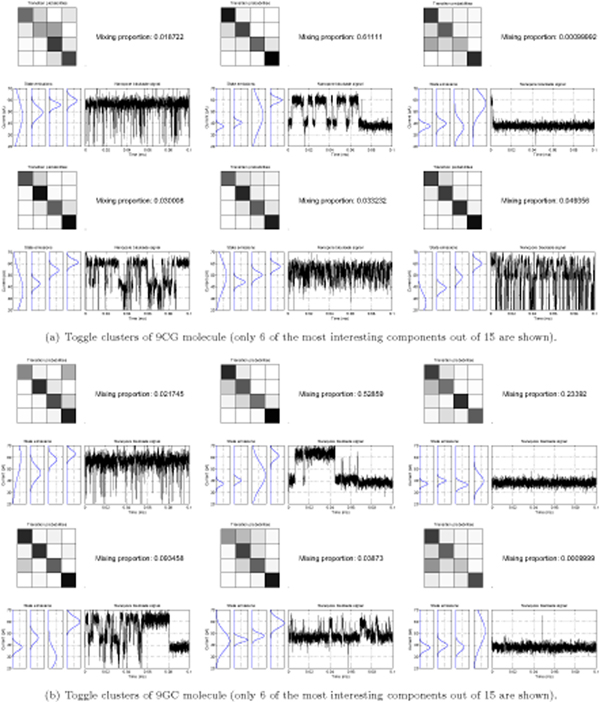
Toggle clusters for 9GC and 9CG molecules. The mixture proportions correspond to the frequency of a certain toggle mode. Sixteen possible transitions corresponding to profile shown in Figure 7 (a) are shown as chessboard, the darker the area of a cell the more probable a transition. Emissions corresponding to each of the four hidden HMM states are shown below the transitions matrix. MAP classified 100 ms toggle sample from the learning set corresponding to a certain profile is also shown.

**Figure 5:**
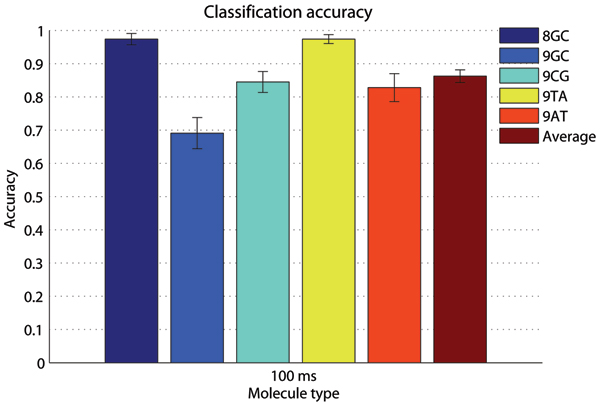
MAP classification accuracy with 10-fold resampling on a split-sample data (with 4 levels and 15 components).

**Figure 6:**
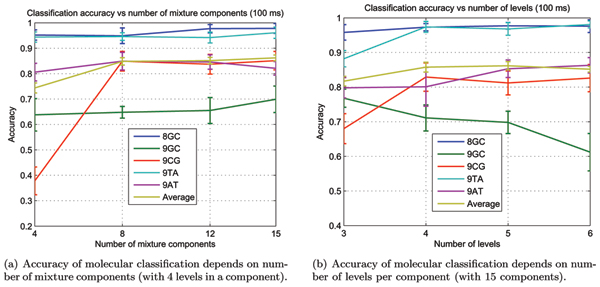
Increasing model complexity affects accuracy.

**Figure 7:**
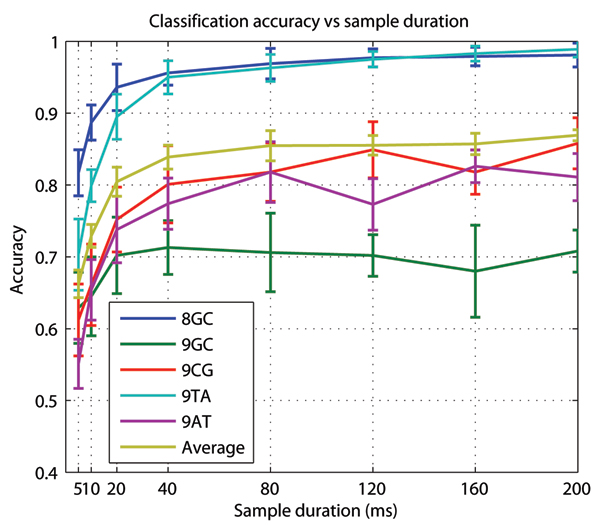
Accuracy of molecular classification depends on sample duration.

We have learned blockade signal clusters for five different types of molecules: two such profile mixtures, learned in 50 iterations, are shown in Figure 4. The classification accuracy is shown in Figure 5, where we used 10-fold resampling of 500 labeled toggle sample subsets from our test set [see Section *Methods*] (the 10-fold resampling is needed to perform majority-vote classification stabilization). The resampling offers a similar stabilization on classifications, and at similar computational expense, to what is done via data-rejection in [5,6]. Accuracy here is defined as

(1) $$Accuracy=\frac{TP+TN}{TP+FP+TN+FN},$$

where True Positives (TP), True Negatives (TN), False Positives (FP) and False Negatives (FN) are among the classified data samples. We have systematically investigated how the model complexity affects accuracy as shown in Figure 6, where average accuracy does not improve for the model of more than 12 components and more than 4 blockade levels, although some individual molecules take advantage of increased model complexity as their classification becomes more accurate. We have also investigated the blockade signal duration needed for proper classification, as shown in Figure 7, and for the data-sets examined found that samples with more that 100 ms duration yield little in either average classification accuracy or classification time. We tried using ionic flow blockade samples of 200 ms in the MHMM training, for example, with no apparent improvement to classification accuracy over the 100 ms duration samples. This behavior was not observed with the non-MAP, large-state (but scalable), approach used in [5,6], where greater observation times led to improved classification (although there is agreement that there was diminishing returns on learning sets for signal durations greater then 100 ms, and, especially, if greater than 500 ms).

**Figure 8:**
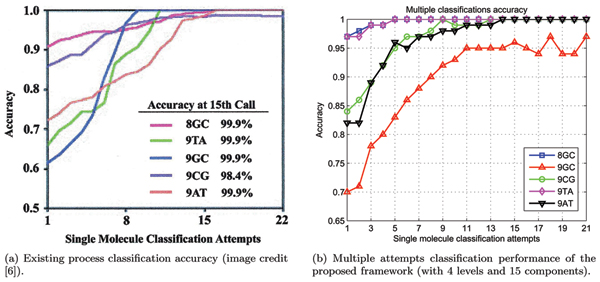
Proposed and existing process classification accuracy.

The accuracy of consecutive same-analyte toggle samples classification is shown in Figure 8(b), where we reach 100% performance within 14 classifications, except for the 9GC molecule, which underperformed when compared with [5,6]. The difficulty with 9GC classification accuracy convergence could be explained by substantial confusion with 9AT toggles, which reaches ~17% at first classification round and reluctantly reduces to ~3% after 21 classification rounds.

The accuracy improvement is consistent with the accuracy of the previously reported classification process [6] as shown in Figure 8(a) (except for the 9GC molecule). The failure to discern 9GC from 9AT in the approach described here, and not in prior efforts [5,6], may simply be the result of better blockade-level resolution 'fine-structure' with the prior model.

The better resolution between 9GC and 9AT channel blockades obtained with the 50-state *single *HMM (used in [5,6]) may simply be due to the fixed 1pA resolution (the state quantization bin-size) providing a critical resolving capability between very similar blockade signals. If true, a hybrid solution may be to directly incorporate fine-structure into the 4-state *multiple *HMM processing model that is used here, by adding *fine-structure *states at 1pA distances on either side of the 4 states identified by EM. Efforts along these lines are ongoing (see *Discussion*).

The MHMM analysis framework first has been implemented in a concurrent fashion on a quad-core Sun Ultra 40 M2 machine with speedup factor 3.66 as compared to a conventional implementation, and then distributed to the five machines of the same type with Java RMI with additional speedup of 4.02, which translates to the total speedup of 3.66 × 4.02 = 14.71.

## Methods

**Figure 9:**
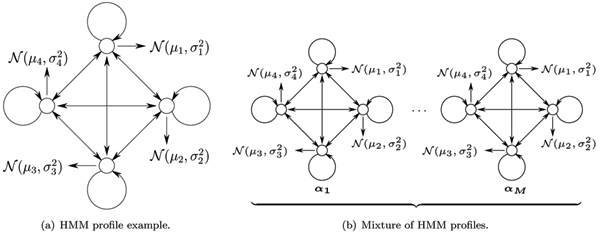
HMM profile and mixture of profiles.

In our approach we used unsupervised distributed learning of nanopore ionic flow blockade toggles with an MHMM. MHMMs have a long record of successful implementations that started in speech recognition [8] and later were used for clustering protein families [9], sequences [10] and in the search for splicing enhancers [11]. We use the HMM profile shown in Figures 2 and 9(a) to model the channel blockade process using MHMM components as shown in Figure 9(b). Justification for using such profiles is provided in [12], where we have found the duration of ionic flow blockade levels to be distributed with a simple geometric distribution. The noise at a fixed-level blockade level is typically found to be Gaussian, consistent with the overall thermal and shot noise background for the transient-binding fixed-flow-geometry environments formed by channel and blockading elements.

The ionic flow blockade records were obtained from the previous studies [6]. Two axon binary files (each containing 500 blockade samples of 300 ms) have been used to learn the probabilistic profiles for each hairpin molecule. The first 100 ms of each channel blockade is the basis of the first test set. Four other axon binary files, with uninterrupted recordings (non-sweep data), for each hairpin molecule and recorded on the same day, are then used for testing. The test set was formed by equiprobable sampling of 500 labeled blockade samples from the pool of test files.

Another test set was constructed from the above data files to measure accuracy of consecutive same-analyte toggle sample classification. In this instance we take all the available blockade signal coming from the test files of a certain molecule (not just the first 100 ms) and use multiple sample draws from the same signal blockade (i.e., consecutive 100 ms segments). With the 100 ms signal samples drawn from the same blockade event, we perform MAP scoring followed by majority vote (with random resolution of ties). (Note: the data rejection employed in [6] could be made roughly equivalent to the signal resampling approach described here by simply collecting consecutive 100 ms samples, as done here, and having classification on a given blockade once the signal isn't rejected, the only difference with the classification post-processing being that in this effort majority vote is employed instead.) Accuracy is calculated as the number of correct classifications matching the known molecule type to the total number of classification events. As in [6], the ionic flow in each record has been normalized to the open channel current 120 pA prior to learning and testing.

For our distributed MHMM system implementation we have used cluster of five workstations Sun Ultra 40 M2, each equipped with two AMD Dual-Core Opteron processors (2220SE 2.8 GHz), connected through gigabit Ethernet switch.

### HMM definition and EM learning

The following parameters describe the conventional HMM implementation according to [13]:

• A set of states *S *= {*S*_1_,..., *S*_*N*_} with *q*_*t *_being the state visited at time *t*,

• A set of PDFs *B *= {*b*_1_(*o*),..., *b*_*N*_(*o*)}, describing the emission probabilities *b*_*j*_(*o*_*t*_) = *p*(*o*_*t*_|*q*_*t *_= *S*_*j*_) for 1 ≤ *j *≤ *N*, where *o*_*t *_is the observation at time-point *t *from the sequence of observations *O *= {*o*_1_,..., *o*_*T*_},

• The state-transition probability matrix A = {*a*_*i*,*j*_} for 1 ≤ *i*, *j *≤ *N*, where *a*_*i*, *j *_= *p*(*q*_*t*+1 _= *S*_*j*_|*q*_*t *_= *S*_*i*_),

• The initial state distribution vector ∏ = {*π*_1_,..., *π*_*N*_}.

A set of parameters *λ *= (∏, *A*, *B*) completely specifies an HMM. Here we describe the HMM parameter update rules for the EM learning algorithm rigorously derived in [14]. When training the HMM using the Baum-Welch algorithm (an Expectation Maximization procedure), first we need to find the expected probabilities of being at a certain state at a certain time-point using the forward-backward procedure as shown in Table 1.

**Table 1:** Forward and backward procedures.

Forward procedure	Backward procedure
*α*_*t*_(*i*) ≡ *p*(*o*_1_,..., *o*_*t*_|*q*_*t *_= *S*_*i*_, *λ*)	*β*_*t*_(*i*) ≡ *p*(*o*_*t*+1_,..., *o*_*T*_|*q*_*t *_= *S*_*i*_, *λ*)
• Initially *α*_1_(*i*) = *π*_*i*_*b*_*i*_(*o*_1_) for 1 ≤ *i *≤ *N*,	• Initially *β*_T_(*i*) = 1 for 1 ≤ *i *≤ *N*,
• $\alpha_{t}(j)=\left[\sum_{i=1}^{N}\alpha_{t-1}(i)a_{i,j}\right]b_{j}(o_{t})$ for t = 2, 3,..., T and 1 ≤ *j *≤ *N*,	• $\beta_{t}(i)=\sum_{j=1}^{N}\alpha_{i,j}b_{j}(o_{t+1})\beta_{t+1}(j)$ for *t *= *T *- 1,...,1 and 1 ≤ *i *≤ *N*,
• Finally $p(O|\lambda )=\sum_{i=1}^{N}\alpha_{T}(i)$ is the sequence *likehood*.	• Finally $p(O|\lambda )=\sum_{i=1}^{N}\pi_{i}b_{i}(o_{1})\beta_{1}(i)$.

Let us define *ξ*_*t*_(*i*, *j*) as the probability of being in state *i *at time *t*, and state *j *at time *t *+ 1, given the model and the observation sequence

(2) $$\xi_{t}(i,j)=p(q_{t}=S_{i},q_{t+1}=S_{j}|O,\lambda )=\frac{\alpha_{t}(i)a_{i,j}b_{j}(o_{t+1})\beta_{t+1}(j)}{p(O|\lambda )},$$

and *γ*_*t*_(*i*) as the probability of being in state *i *at time *t*, given the observation sequence and the model

(3) $$\gamma_{t}(i)=p(q_{t}=S_{i}|O,\lambda )=\frac{\alpha_{t}(i)\beta_{t}(i)}{\sum_{i=1}^{N}\alpha_{t}(i)\beta_{t}(i)}=\sum_{j=1}^{N}\xi_{t}(i,j).$$

The HMM maximization step using these probabilities is shown in Table 2.

**Table 2:** Maximization step in HMM learning.

Initial probability estimate	Transition probability estimate	Emission parameters estimate
${\hat{\pi }}_{i}$ = *γ*_1_(*i*), for 1 ≤ *i *≤ *N*.	${\hat{a}}_{i,j}=\frac{\sum_{t=1}^{T-1}\xi_{t}(i,j)}{\sum_{t=1}^{T-1}\gamma_{t}(i)}$, for 1 ≤ *i*, *j *≤ *N*.	Gaussian emission ${\hat{b}}_{j}(o)\to \mu =\frac{\sum_{t=1}^{T}o_{t}\gamma_{t}(j)}{\sum_{t=1}^{T}\gamma_{t}(j)}$, ${\hat{b}}_{j}(o)\to \sigma^{2}=\frac{\sum_{t=1}^{T}{\left(o_{t}-{\hat{\mu }}_{j}\right)}^{2}\gamma_{t}(j)}{\sum_{t=1}^{T}\gamma_{t}(j)}$, for 1 ≤ *j *≤ *N*.

### EM learning of HMM mixture

The objective of mixture learning is to maximize the likelihood function $p(O|\Theta )=\Pi_{i=1}^{N}p(o_{i}|\Theta )=ℒ(\Theta |O)$, i.e. we wish to find the locally optimal set of parameters $\Theta^{*}=argmaxℒ(\Theta |O)$ by using the Expectation Maximization (EM) iterative procedure and the set of data points O.

The Expectation step in the mixture fitting algorithm is done by computing the responsibility matrix of the components given the data points:

$$\underset{M\text{ mixture components}}{\underset{︸}{\begin{array}{ccc} p(\lambda_{1}|O_{1},\Theta ) & \cdots & p(\lambda_{M}|O_{1},\Theta )\\ p(\lambda_{1}|O_{2},\Theta ) & \cdots & p(\lambda_{M}|O_{2},\Theta )\\ p(\lambda_{1}|O_{3},\Theta ) & \cdots & p(\lambda_{M}|O_{3},\Theta )\\ \cdots & \cdots & \cdots \\ p(\lambda_{1}|O_{K},\Theta ) & \cdots & p(\lambda_{M}|O_{K},\Theta ) \end{array}}}\}K\text{ data points}$$

We use Bayes' rule to find the posterior probability (responsibility) of a mixture component with parameters *λ*_*m *_and emission sequence *O*_*k*_:

(4) $$p(\lambda_{m}|O_{k},\lambda )=\frac{\alpha_{m}p(O_{k}|\lambda_{m})}{\sum_{j=1}^{M}\alpha_{j}p(O_{k}|\lambda_{j})}.$$

The Expectation step is followed by the maximization step where we re-estimate parameters.

• Mixture proportions

(5) $${\hat{\alpha }}_{m}=\frac{1}{K}\sum_{k=1}^{K}p(\lambda_{m}|O_{k},\lambda ),$$

• Initial probabilities

(6) $${\hat{\Pi }}_{m}=\frac{\sum_{k=1}^{K}{\hat{\Pi }}_{m}^{k}p(\lambda_{m}|O_{k},\lambda )}{\sum_{k=1}^{K}p(\lambda_{m}|O_{k},\lambda )},$$

where ${\hat{\Pi }}_{m}^{k}$ is an estimate of initial probabilities for the component *m *given sequence *O*_*k*_,

• Transitions

(7) $${\hat{A}}_{m}=\frac{\sum_{i=1}^{K}{\hat{A}}_{m}^{k}p(\lambda_{m}|O_{k},\lambda )}{\sum_{k=1}^{K}p(\lambda_{m}|O_{k},\lambda )},$$

where ${\hat{A}}_{m}^{k}$ is an estimate of transition probabilities for the component *m *given sequence *O*_*k*_,

• Emissions

(8) $${\hat{B}}_{m}=\frac{\sum_{k=1}^{K}{\hat{B}}_{m}^{k}p(\lambda_{m}|O_{k},\lambda )}{\sum_{k=1}^{K}p(\lambda_{m}|O_{k},\lambda )},$$

where ${\hat{B}}_{m}^{k}$ is an estimate of emission parameters for the component *m *given sequence *O*_*k*_.

### Distributed EM implementation

As discussed in [15], the computational gain of a *parallel *implementation can greatly depend on model topology. In the speech recognition community researchers are able to use a highly parallel HMM architectures for phoneme and dictionary word recognition. Typically, when a large number of Processing Elements (PEs) is used, the utilization of each element drops due to communication overheads. Therefore, the communication overhead in any parallel architecture must be strictly managed, ideally reduced to a constellation of PEs with shared memory [15]. In recent work [16] we describe the performance of the following HMM EM algorithms (where we studied the last on the list):

• Conventional EM due to Leonard E. Baum and Lloyd R. [17] takes *O*(*T N*) memory and *O*(2*T N Q*_*max *_+ *T *(*Q *+ *E*)) time, where *T *is the length of the observed sequence, *N *is the number of HMM states, *Q*_*max *_is the maximum HMM node out-degree, *E *is the number of free emission parameters, *Q *is the number of free transition parameters.

• Checkpointing EM [18-20] takes *O*($\sqrt{T}$*N*) memory and *O*(3*T N Q*_*max *_+ *T *(*Q *+ *E*)) time,

• Linear memory EM [16,21] takes only *O*(*N*(*Q *+ *E D*)) memory and *O*(*T NQ*_*max*_(*Q *+ *E D*)) time.

Similar improvements are also described for the HMM Viterbi implementation in linear memory [16]. In actual usage with the comparatively small durations generally examined, the checkpointing algorithm was found to be the most memory efficient.

### Distributed checkpointing algorithm for learning from large data samples

**Figure 10:**
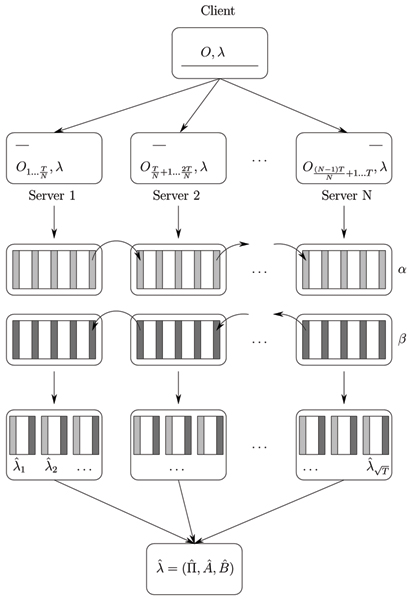
Distributed Checkpointing algorithm.

The distributed checkpointing EM algorithm is shown in Figure 10. Here are the steps in our distributed checkpointing algorithm implementation:

1. Client machine splits data sequence *O *into subsequences *O*_1_,..., *O*_*t*_,..., $O_{\sqrt{T}}$ each of size $\sqrt{T}$ and distributes them across the servers along with *λ*,

2. Find Forward and Backward $\sqrt{T}$ checkpoints in sequential manner at the corresponding servers where emission matrices for *O*_*t *_were calculated and stored,

3. Reconstruct dynamic programming tables of size *N*$\sqrt{T}$ at the servers according to locally stored checkpoints to make local parameter estimate ${\hat{\lambda }}_{t}=({\hat{\Pi }}_{t},{\hat{A}}_{t},{\hat{B}}_{t})$,

4. After calculating local parameter estimate, communicate ${\hat{\lambda }}_{t}$ back to the client machine and calculate $\hat{\lambda }=(\hat{\Pi },\hat{A},\hat{B})$,

5. Redistribute newly found $\hat{\lambda }$ among the server machines for another EM round.

### Distributed MHMM parameter estimate

An MHMM can easily split the responsibilities calculation between several cluster nodes with minimum communication overhead in the following way:

1. For each parameter *λ*_1_,..., *λ*_*m*_,..., *λ*_*M *_and sequence *O*_1_,..., *O*_*k*_,..., *O*_*K *_calculate likelihood *p*(*O*_*k*_| *λ*_*m*_) on the server nodes and communicate them back to the client,

2. Client finds responsibilities for each mixture component and a sequence according to formula (4),

3. Estimated mixture proportions ${\hat{\alpha }}_{1},...,{\hat{\alpha }}_{m},...,\alpha_{M}$ are found on a client node according to (5),

4. The server nodes find ${\hat{\lambda }}_{m}^{k}$ estimates for parameter *λ*_*m *_and sequence *O*_*k *_and send them back to the client,

5. On the client node these newly computed parameters are weighted according to responsibilities (6), (7), (8),

6. Newly found HMM parameters ${\hat{\lambda }}_{1},...,{\hat{\lambda }}_{M}$ are disbursed back to the server nodes for the next round of EM training.

## Discussion and conclusion

There are several advantages in our approach:

• Classification is highly accurate with no data dropped from consideration,

• Model parameters may have intuitive physical interpretation (but not in this study),

• The MHMM implementation is distributed, such that:

- Learning can take a larger number of samples (for improved accuracy),

- Enables real-time analyte classification, currently takes only 0.411 sec to classify 100 ms sample,

- Checkpointing algorithm keeps the memory profile low both on server and client sides without compromising the running time [16].

The need for using a mixture model beyond a simple HMM comes from the observation that generally no more than half of hairpin blockades come from the same mode of hairpin molecule interacting with nanopore (the modes correspond to principal components in the channel blockade stationary statistics profile). Other mode contributions require different probabilistic profiles for classification which naturally leads to a mixture analysis problem. The method shown in Figure 3 doesn't introduce such modes at the HMM-processing stage, relying instead on the strengths of the SVM classifier directly.

Increasing EM-learning model complexity beyond 4 levels and 12 mixture components increases the log-likelihood of fully trained model, but does not lead to better prediction accuracy as shown in Figure 6. The likelihood increase is caused by the model overfitting the data. Overfitting with HMM-profile models, however, isn't found to be as detrimental to the generalization performance as with other learning methods – the main penalty is that the learning and classification times increase dramatically, as we need to estimate progressively increasing number of parameters.

Since we did not computationally exhaust all the possible parameter settings (number of components, number of levels and sample duration), we provide a rationale for the parameter choice we believe is optimal. With preliminary experiments learning on 9CG toggle samples with MHMM of 15 toggle clusters we have consistently exhausted the number of components, many of them converging to the same simple blockade as shown in figure 4(a) at the top right. This observation prompted us to use no more than 12 components in the channel blockade signal-mode mixture model.

The number of four blockade levels corresponds to the physical model of DNA hairpin interacting with nanopore [5]. From the physical perspective the hairpin molecule can undergo different modes of capture blockade, such as Intermediate Level (IL), Upper Level (UL), Lover Level (LL) conductance states and spikes (S) [6]. When a 9 bp DNA hairpin initially enters the pore, the loop is perched in the vestibule mouth and the stem terminus binds to amino acid residues near the limiting aperture. This results in the IL conductance level. When the terminal basepair desorbs from the pore wall, the stem and loop may realign, resulting in a substantial current increase to UL. Interconversion between the IL and UL states may occur numerous times with UL possibly switching to the LL state. This LL state corresponds to binding of the stem terminus to amino acids near the limiting aperture but in a different manner from IL. From the LL bound state, the duplex terminus may fray, resulting in extension and capture of one strand in the pore constriction resulting into short term S state. The allowed transition events between the levels *IL *⇔ *UL *⇔ *LL *⇔ *S *to happen at any time during the analysis procedure. The spikes model, as described in [16], could possibly be used to increase prediction accuracy. However, with the scenario discussed in this manuscript use of such additions did not lead to higher performance since the primary blockade modes shown in Figures 4(a) and 4(b) are void of spikes.

A demo program implementing distributed MHMM analysis framework is available free of charge on our web site .

## Competing interests

The authors declare that they have no competing interests.

## Authors' contributions

AC conceptualized the project, implemented and tested the MHMM EM algorithm for nanopore ionic flow analysis. SWH helped with writing the manuscript and provided many valuable suggestions directing the study. All authors read and approved the final manuscript.
